# Ventriculoperitoneal shunts in non-HIV cryptococcal meningitis

**DOI:** 10.1186/s12883-018-1053-0

**Published:** 2018-05-01

**Authors:** Jia Liu, Zhuo-lin Chen, Min Li, Chuan Chen, Huan Yi, Li Xu, Feng Tan, Fu-hua Peng

**Affiliations:** 1 Department of Neurology, the Third Affiliated Hospital of Sun Yat-Sen University, 600# Tianhe Road, Guangzhou, 510630 Guangdong China; 2 Department of Neurosurgery, the Third Affiliated Hospital of Sun Yat-Sen University, Guangzhou, 510630 Guangdong China; 3 Department of Neurology, Foshan Chinese Medicine Hospital, Foshan, 528000 Guangdong China

**Keywords:** Ventriculoperitoneal shunts, HIV, Cryptococcal meningitis, Ventriculomegaly, Intracranial hypertension

## Abstract

**Background:**

Persistent and uncontrollable intracranial hypertension (ICH) and difficulty in reducing Cryptococcus count are severe problems in cryptococcal meningitis (CM) patients. The therapeutic effects of ventriculoperitoneal shunts (VPS) in non-HIV CM patients are not fully known, and the procedure is somewhat unusual. Here, our study offers a review to investigate the role of VPS in non-HIV CM.

**Methods:**

We retrospectively collected data on 23 non-HIV CM patients with and without ventriculomegaly from 2010 to 2016. Their demographic data, clinical manifestations, cerebrospinal fluid (CSF) features and outcomes were analysed.

**Results:**

We found that non-HIV CM patients without ventriculomegaly were older, had earlier treatment times and had shorter symptom durations than CM patients with ventriculomegaly. In both groups, headache, vomiting, fever and loss of vision were the most common clinical features. CSF pressure and Cryptococcus count were significantly decreased after operation. VPS could provide sustained relief from ICH symptoms such as headache. 13% of patients had poor outcomes because of serious underlying disease, while 87% of patients had good outcomes.

**Conclusions:**

The use of a VPS is helpful in decreasing ICH and fungal overload in non-HIV CM patients, and VPS should be performed before CM patients present with symptoms of severe neurological deficit.

## Background

*Cryptococcus neoformans* is an opportunistic pathogen and is an important cause of fungal meningitis associated with high morbidity and mortality rates. It commonly occurs in immunosuppressed patients with HIV infections or transplant conditioning, as well as in previously healthy individuals [1, 2]. There are many patients without a severe immunocompromising illness before the development of cryptococcal meningitis (CM) who have a negative HIV test result. Non-HIV related cryptococcosis is becoming a more significant subpopulation in the developed world, accounting for approximately one-third of cases [3]. Therefore, an increased recognition of CM in immunocompetent hosts has begun to direct more attention towards this infection [4]. Persistent and uncontrollable intracranial hypertension (ICH) is a severe complication in patients with CM and is closely related to the count of Cryptococcus. Delays in treatment of ICH are directly related to poor outcomes, including severe headache, papilledema, loss of vision and hearing, and disturbances in consciousness [5]. ICH is an important risk factor for neurological deficits in CM patients and is associated with early death [6, 7]. It has been documented that reducing ICH to normal levels is essential to improving CM patients’ prognosis [6, 8]. Many researchers have previously shown that ventriculoperitoneal shunt (VPS) placement in HIV-infected patients with ICH can result in a good response and a positive outcome [9–11]. In HIV-related CM patients, the uncontrollable ICH could be relieved by VPS [12]. Nevertheless, few studies have examined VPS in non-HIV CM patients. Some researchers have suggested that the effects of VPS in CM patients with hydrocephalus were limited and that this treatment could not be used effectively in comatose patients [7]. However, Po-Chou Liliang et al. proposed that uncontrollable ICH in non-HIV CM patients without ventriculomegaly could be resolved by the use of VPS, and the patients’ neurological deficits could be improved [13]. Hence, the proposed role of VPS in non-HIV CM patients has also been inconsistent.

Here, our study offers a retrospective review to analyse the difference after VPS between non-HIV CM patients with and without ventriculomegaly. We conducted this study to assess the predictive value of the response to VPS in non-HIV CM patients. An additional aim was to evaluate whether there were differences in the effects of VPS in CM patients with and without ventriculomegaly.

## Methods

### Clinical data collection

**Fig. 1:**
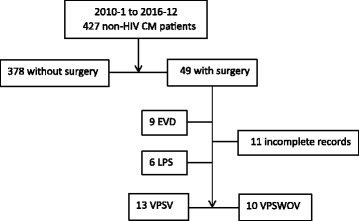
Enrolment process of patients. CM: cryptococcal meningitis; EVD: external ventricular drainage; LPS: lumboperitoneal shunt; VPS: ventriculoperitoneal shunts; VPSV: VPS with ventriculomegaly; VPSWOV: VPS without ventriculomegaly

**Fig. 2:**
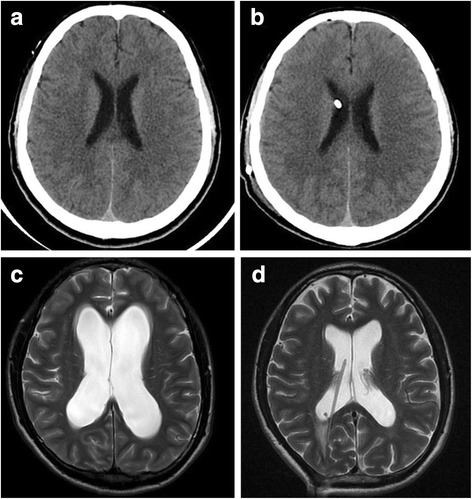
**a** and **b** Head CT of a 40-year-old VPSWOV patient before and after operation. **a** Pre-operative CT shows no dilation of the cerebral ventricles (**b**) postoperative CT shows no dilation of the cerebral ventricles. **c** and **d** Head MR of a 28-year-old VPSV patient before and after operation. **a** Pre-operative axial T2-weighted image shows dilation of the cerebral ventricles (**b**) postoperative T2-weighted shows narrowing of the cerebral ventricles

This was a retrospective study. The patients were from the Third Affiliated Hospital of Sun Yat-sen University, Guangzhou, China. From January 2010 to June 2016, the clinical data of 427 non-HIV infected CM patients were reviewed. A total of 378 patients without surgery were excluded. A total of 23 patients satisfied the diagnostic criteria and were recruited into our study. The details of the enrolment process are presented in Fig. 1. The typical brain images of patients with ventriculomegaly or without were shown in Fig. 2.

### Patients’ definitions

CM patients were identified according to symptomatology along with isolation of *C. neoformans* in cerebrospinal fluid (CSF) culture or a positive result of CSF India ink microscopy [14].

Radiographic diagnosis of ventriculomegaly was made based on enlargement of the temporal horn of the lateral ventricle, without obvious brain atrophy on the initial and/or follow-up CT or MRI [7, 10, 11].

Demographic data, risk factors, time from diagnosis to shunt, clinical features before and after VPS, CSF characteristics, CT/MR findings, antifungal therapy and outcomes were recorded (Tables 1 and 2).

**Table 1:** Demographic and clinical features in VPSV and VPSWOV patients

No	Age/ gender	Risk factors	Time from diagnosis to shunt (m)	neurological deficit		antifungal therapy	Outcome 1 m
				*Before VPS*	*After VPS improved*		
				VPSV			
1	38/M	Hepatitis B	4	H,VL,HL	H	AMBisome+Fluc+5FC	better
2	36/M	Hepatitis B, Liver cirrhosis	1.5	H,VL,LOC	H,VL,LOC	AMB + Fluc+5FC	worse
3	40/M	Diabetes mellitus	3	H/V	H	AMB + Fluc+5FC	better
4	17/M		3.5	H/V,VL,LOC	H/V,VL,LOC	AMB + Fluc+5FC	better
5	28/M		1.5	H/V,VL	H,VL	Fluc+5FC	better
6	53/M		6	V, LOC	V, LOC	AMB + Fluc+5FC	better
7	27/M	pulmonary cryptococcosis	3	H/V,VL	H/V	AMB + Fluc+5FC	better
8	21/M		5	H	H	AMB + Fluc+5FC	better
9	31/M	Hepatitis B, Diabetes mellitus	6	H,VL	H,VL	Fluc+5FC	better
10	19/M	AIHA	4	H/V,CO	H/V,CO	AMB + Fluc+5FC	better
11	18/M	Hepatitis B	2	H/V	H/V	AMB + Fluc+5FC	better
12	53/F		3.5	V,LOC	V,LOC	AMB + Fluc+5FC	better
13	50/M	Hepatitis B	6	H,VL	H	Fluc+5FC	better
				VPSWOV			
14	41/F	pulmonary cryptococcosis	4	H/V	H	AMB + Fluc+5FC	better
15	35/M		3	H/V,VL,LOC	H/V,VL,LOC	AMB + Fluc+5FC	better
16	40/M	pulmonary cryptococcosis	3	H/V,VL,HL	H/V,VL	AMB + Fluc+5FC	better
17	51/M	Diabetes mellitus	0.5	H/V,VL	H/V,VL	AMB + Fluc+5FC	better
18	41/M		2	H,VL, CO	H,VL	AMB + Fluc+5FC	better
19	61/F	Malignancy	4	H/V	H/V	Fluc+5FC	worse
20	37/M	Hepatitis B	2	H/V,VL,HL	H/V,VL	AMB + Fluc+5FC	better
21	55/F	SLE	0.5	H/V,VL	H/V,VL	Fluc+5FC	better
22	17/M	pulmonary cryptococcosis	1	H/V,LOC	H/V,LOC	AMB + Fluc+5FC	better
23	45/F	AIHA	1	H,VL,HL,LOC	H,VL	AMB + Fluc+5FC	NA

*AIHA* autoimmune haemolytic anaemia, *SLE* systemic lupus erythematosus, *H/V* headache and vomiting, *VL* visual loss, *HL* hearing loss, *LOC* loss of consciousness, *CO* convulsions, *NA* Not available, *AMB* Amphotericin B, *5FC* flucytosine, *Fluc* Fluconazole, *M* month, 1–13: VPSV, 14–23: VPSWOV

**Table 2:** Clinical and CSF characteristics in VPSV and VPSWOV patients pre-operation

Characteristic	VPSV (*n* = 13)	VPSWOV (*n* = 10)	*P*
Sex(M/F)	12/1	6/4	0.127
Age(Y) median (range)	28(17–53)	41(17–61)	0.032
Symptoms duration, days, median (range)	90 (20–180)	30(10–180)	0.019
headache	11/13 (84.6%)	10(100%)	0.486
fever	7/13(53.8%)	5/10(50%)	>0.999
Vomiting	8/13(61.5%)	8/10(80%)	0.405
Visual symptoms	7/13(53.8%)	7/10(70%)	0.669
Auditory symptom	2/13(15.4%)	3/10(30%)	0.618
Altered mental status	5/13(38.5%)	3/10(30%)	>0.999
Seizure	1/13(7.69%)	2/10(20%)	0.560
Indian ink smear (+)	12/13(92.3%)	10/10(100%)	>0.999
CSF culture positive (+)	8/13(61.5%)	7/10(70%)	>0.999
Time to operation(m)	3.5(1.5–6)	2(0.5–4)	0.028
CSF pressure (mmH2O) median (range)	330(200–1020)	330(204–660)	0.059
WBC count(×106/l) median (range)	60(0–550)	36(1–496)	0.225
Protein, g/L median (range)	0.53(0.12–4.06)	0.8(0.23–1.62)	0.139
Glucose(mmol/l) median (range)	2.33(0.01–5.58)	2.53(0.23–5.7)	0.753
Chloride(mmol/l) median (range)	118(103–133)	120(108–136)	0.150
India ink Cryptococcus count	23(0–1.3E4)	1.94E3(4–1.67E5)	0.001

*M* male, *F* female, *CSF* cerebrospinal fluid, *P* VPSV versus VPSWOV pre-operation

### Laboratory measurement

Repeated lumbar puncture (LP) was performed in all the patients, and CSF open pressure, differential counts, glucose, protein, chloride, India ink smear and CSF culture were recorded. The CSF sample was subjected to India ink preparation. Cryptococcus count was determined by counting the number of *Cryptococcus neoformans* per millilitre of CSF via microscopic examination after India ink test. Brain computed tomography (CT) and/or magnetic resonance imaging (MRI) was performed before and after operation. All images were analysed by experienced neuroradiologists.

### Therapeutic methods

All patients received antifungal therapy with the following methods: amphotericin B (AMB): 0.7–1.0 mg/kg·d, fluconazole (Fluc): 400–800 mg/d, flucytosine (5FC): 100 mg/kg·d [15]. Patients underwent LP three or four times within 1 week, according to their condition. After they had received routine VP operations, LP was performed to dynamically monitor the CSF changes. All patients received follow-up care 1 month after discharge.

### Statistical analysis

All statistical analyses in this study were performed using SPSS (version 16.0, Chicago, IL, USA). Numerical variables were presented as the mean ± standard deviation (SD) or median (interquartile range), and categorical variables were expressed as a percentage. Statistical significance was set at *P* < 0.05. Student’s t-test was used to analyse normally distributed variables. A mean of repeated-measure ANOVA was used for continuous variables (e.g., CSF parameters). The nonparametric Mann-Whitney *U* test was used for non-normally distributed data. Chi-square or Fisher’s exact test was performed for categorical variables.

## Results

### Subject clinical characteristics

A cohort of 23 non-HIV CM patients who were treated with VPS, including 13 with ventriculomegaly (VPSV) [12 males and 1 female] and 10 without ventriculomegaly (VPSWOV) [6 males and 4 females], were enrolled in our study (Fig. 1 and Table 2). There was no significant difference in gender composition between the groups. The post-operation period was divided into three stages: within 1 week, when discharged from hospital and re-hospitalization 1 month later.

### The clinical and demographic features of the subjects were summarized in Tables 1 and 2

The male sex was predominant in both groups. Most of the patients had predisposing factors such as liver disease, diabetes mellitus, pulmonary cryptococcosis, malignancy or autoimmune disease. Compared with the VPSV group, those with VPSWOV were older (mean age: 41 years vs. 28 years, *p* = 0.032), and with shorter symptom durations (30 vs. 90 days, *p* = 0.019) and earlier surgical treatments (2 vs. 3.5 months, *p* = 0.028). All the patients had some symptoms of neurological deficit, including headaches, vomiting, vision and hearing loss, loss of consciousness and convulsions. Among these symptoms, headache, vomiting, fever and loss of vision were the most common clinical features in both the VPSV and VPSWOV groups. In our observation period, one male VPSV patient and two VPSWOV patients’ conditions deteriorated due to their serious underlying diseases (case 2, 19 and 23).

### Comparisons of CSF characteristics between VPSV and VPSWOV groups before operation

As shown in Table 2, in both groups, ICH during LP and CSF assays were abnormal in all patients. High pleocytosis, protein levels, and fungal overload along with low glucose and chloride levels were detected. Higher fungal load was observed in the VPSWOV group than in the VPSV group (*p* = 0.001) in the India ink staining test. However, no intergroup differences were found in CSF cell count or protein, glucose or chloride levels.

### Observed clinical and CSF features in VPSV and VPSWOV pre-operation and 1 week post-operation

Within 1 week after VPS, we found that headache, vision, hearing and consciousness were improved to different degrees in both VPSV and VPSWOV patients, and headache had improved significantly (*p* < 0.01). Moreover, ICH in both groups was significantly decreased after operation when compared with pre-operation (153 vs. 330, 180 vs. 330, *p* < 0.001; Table 3). No significant difference was found between the two groups (*p* = 0.402). Patients with VPSV (0 vs. 23) had a significantly lower Cryptococcus count after the operation than before (*p* = 0.014), and patients with VPSWOV also had a lower fungal count after the operation (3.12E2 vs. 1.94E3, *p* = 0.337), although the differences were not statistically significant.

**Table 3:** Comparison of clinical and CSF characteristics in VPSV and VPSWOV patients within 1 week after the operation

		VPSV (*n* = 13)	VPSWOV (*n* = 10)	*P*_1_	*P*_2_	*P*
symptoms improved	headache	10/11(90.1%)	10/10(100%)	0.001	<0.001	>0.999
	Visual	4/7(57.1%)	5/7(71.4%)	>0.999	0.153	>0.999
	Auditory	1/2(50%)	0/3	0.371	0.07	0.40
	Mental change	4/5(80%)	2/3(66.7%)	0.615	0.510	0.464
CSF	CSF pressure (mmH2O) median (range)	153(55–330)	180(95–330)	<0.001	<0.001	0.402
	WBC count (×106/l) median (range)	70(2–459)	80(25–232)	0.42	0.018	0.868
	Protein, g/L median (range)	1.43(0.3–7.7)	1.61(0.51–3.98)	<0.001	<0.001	0.334
	Glucose(mmol/l) median (range)	1.7(0.03–3.54)	1.17(0.43–7.45)	0.008	0.026	0.526
	Chloride(mmol/l) median (range)	117(103–126)	116(103–126)	0.285	0.020	0.96
	India ink Cryptococcus count	0(0–480)	3.12E2(0–3.01E4)	0.014	0.337	0.003

*P*_1_ pre-operation versus within 1 week post operation in the VPSV group

*P*_2_ pre-operation versus within 1 week post operation in the VPSWOV group

*P* VPSV versus VPSWOV within 1 week post operation

### Outcomes and follow-up

We further assessed outcomes for both groups at discharge and at the follow-up re-hospitalization 1 month later. Details of the clinical characteristics and laboratory examinations are shown in Tables 4 and 5. We were able to observe that VPSV patients had significantly decreased ICH (100 vs. 330, 158 vs. 330, *p* < 0.001) and obviously reduced fungal load (0 vs. 23, *p* < 0.01) regardless of when they were discharged or re-hospitalized 1 month later. Interestingly, we could reach a similar conclusion in the VPSWOV group, where we found that at these same time points, patients also had significantly lower ICH (142 vs. 330, 125 vs. 330, *p* < 0.001) and fungal load (0 vs. 1.94E3, *p* < 0.01). There was no evidence to show that there were differences in ICH and Cryptococcus count between the VPSV and VPSWOV groups.

**Table 4:** The conditions of VPSV and VPSWOV patients when they were discharged from hospital

		VPSV (*n* = 13)	VPSWOV(*n* = 10)	*P*_1_	*P*_2_	*P*
symptoms improved	headache	11/11(100%)	9/10(90%)	<0.001	<0.001	0.476
	Visual	5/7(71.4%)	6/7(85.7%)	0.374	0.05	>0.999
	Auditory	1/2(50%)	2/3(66.7%)	0.371	0.51	>0.999
	Mental change	4/5(80%)	2/3(66.7%)	0.615	0.510	0.464
CSF	CSF pressure (mmH2O) median (range)	100(50–200)	142(90–330)	<0.001	<0.001	0.064
	WBC count (×106/l) median (range)	26(6–70)	80(25–232)	0.62	0.798	0.281
	Protein, g/L median (range)	1.27(0.18–4.8)	1.61(0.51–3.98)	0.018	0.002	0.325
	Glucose(mmol/l) median (range)	1.61(0.69–5)	1.17(0.43–7.45)	0.04	0.312	0.806
	Chloride(mmol/l) median (range)	124(120–128)	122(115–132)	0.03	0.935	0.166
	India ink Cryptococcus count	0(0–32)	0(0–5)	0.001	<0.001	0.577

*P*_1_ pre-operation versus discharged from hospital in the VPSV group

*P*_2_ pre-operation versus discharged from hospital in the VPSWOV group

*P* VPSV versus VPSWOV at discharge from hospital

**Table 5:** Follow up with VPSV and VPSWOV patients when they returned to hospital 1 month after the operation

		VPSV (*n* = 13)	VPSWOV (*n* = 9)	*P*_1_	*P*_2_	*P*
symptoms improved	headache	12/13(92.3%)	8/9(88.9%)	0.001	<0.001	>0.999
	Visual	5/7(71.4%)	5/6(83.3%)	0.374	0.05	>0.999
	Auditory	1/2(50%)	2/3(66.7%)	0.371	>0.999	>0.999
	Mental change	4/5(80%)	1/2(50%)	0.615	0.364	0.333
CSF	CSF pressure (mmH2O) median (range)	158(65–280)	125(50–180)	<0.001	<0.001	0.509
	WBC count (×106/l) median(range)	14.5(8–46)	21(2–86)	0.014	0.185	0.488
	Protein, g/L median (range)	0.95(0.15–3.86)	1.31(0.18–2.16)	0.236	0.096	0.692
	Glucose(mmol/l) median (range)	2.25(1.24–2.89)	2.42(1.27–4.17)	0.384	0.593	0.644
	Chloride(mmol/l) median (range)	124(114–131)	124(112–130)	0.104	0.264	0.742
	India ink Cryptococcus count	0(0–266)	0(0–5)	0.004	<0.001	0.377

*P*_1_ pre-operation versus returned to hospital after 1 month in the VPSV group

*P*_2_ pre-operation versus returned to hospital after 1 month in the VPSWOV group

*P* VPSV versus VPSWOV when patients returned to hospital 1 month after the operation

Of all symptoms, headache was most obviously improved after surgery, and this improvement was stable during our observation period (*p*<0.01). Nevertheless, some patients with vision and hearing symptoms were not reversible due to the long-term ICH. Additionally, we found that most of patients had good outcomes. The exceptions were cases 2, 19 and 23, who had a poor prognosis after the operation, and case 23, which was missing from follow-up.

### Analysis of the cases with poor patient outcomes

Case 2, a 36-year-old male with VPSV, presented with vomiting and loss of vision and hearing and was unconscious. Before he received VPS, LP indicated 400 mmH_2_O intracranial pressure (ICP) and 7500 mL^− 1^ Cryptococcus count. Medical history showed that he had hepatitic cirrhosis. After VPS, his vomiting and consciousness state were improved. Vision and hearing had no obvious improvement. As the illness progressed, his liver function deteriorated and he had poor outcome.

Case 19 was a 61-year-old female with VPSWOV and uterine cervical cancer. After VPS, her headache was improved, but she had a poor outcome because the cancer had metastasized to other parts of the body.

Case 23 was a 45-year-old female VPSWOV patient with autoimmune haemolytic anaemia (AIHA). She had been treated with hormones for a very long time. After VPS, her headache and vomiting were improved, but she had a poor prognosis.

In summary, the poor outcomes for each of these patients were closely related to serious underlying diseases, not shunt-related complications.

## Discussion

CM is the most common fungal meningitis in HIV-positive patients. In the absence of HIV, CM patients with underlying immune dysfunction, such as organ transplantation, corticosteroid medication use and malignancy, account for the majority of cases. In our study, we found that liver disease, diabetes mellitus, malignancy, pulmonary cryptococcosis and autoimmune diseases were the major underlying diseases. These findings had some similar features to a previous study in HIV-negative patients [16, 17] but also showed some differences. In our study, we found that in the VPSV group, 5 of the 13 (38.5%) patients suffered from liver diseases. Five patients (38.5%) had no apparent underlying disease. In the VPSWOV group, 3 of the 10 patients (30%) had pulmonary cryptococcosis. Two patients (20%) had no apparent underlying disease. The male sex was predominant in both groups. VPSWOV patients were obviously older than VPSV, and people with VPSWOV tended to receive surgical treatment earlier, making the symptom duration shorter.

Uncontrollable ICH, a severe complication in patients with CM, is defined as a CSF opening pressure > 250 mmH_2_O and is generally considered to relate to high fungal burden in the CSF [18]. Symptoms include headache, papilledema, vomiting, vision and hearing loss, and impaired mentation. Our study showed that headache, vomiting, fever and loss of vision were the most common clinical features in both the VPSV and VPSWOV groups. Headache could continue to ease after VPS operation. On the other hand, impaired eyesight and hearing, as well as altered mental status, were not improved significantly.

An elevated CSF pressure level is significantly linked to increased mortality and poor outcomes [19, 20]. The goal of CM treatment is to reduce fungal burden and to prevent long-term neurological deficits. The decompression is a very important parameter in this management. Ventriculomegaly is an uncommon complication of CM patients. There are multiple treatments to control ICP such as medications, repeated LP, temporary external drainage or permanent VPS [15]. For CM patients with progressive ventriculomegaly, VPS is a favoured method of diversion to relieve the ICH, which is well studied in HIV-positive [10, 21] and HIV-negative [7, 11] CM patients. However, relevant articles about VPS treatment in non-HIV patients without ventriculomegaly are limited. Available data are limited to case reports [13] and smaller case studies [22]. Comparative data concerning the non-HIV CM population with and without ventriculomegaly are sparse. Here, we present a comprehensive analysis of clinical, CSF characteristics and prognosis in both groups in order to further assess the roles of VPS in non-HIV CM patients.

The mechanisms of the development of elevated ICP with ventriculomegaly in CM are complex. Some reports have shown the following possible pathogenesis pathways: (1) the channels for CSF drainage may be blocked by fungal polysaccharides, or (2) the passage of CSF across the arachnoid villi may be blocked by an inflammatory response [23]. For those CM patients with ICH but without ventriculomegaly, previous research has suggested that (1) deposits of cryptococcal capsular polysaccharides on the brain surface and within the parenchyma tissue could lead to the brain’s inability to respond to the increased CSF volume and pressure [7, 24]. (2) The venous stenosis may limit cerebral blood outflow due to an infection-mediated mechanism [21]. In brief, ICH is closely associated with fungal overload. In our study, by comparing the pre-operation CSF characteristics, we found that a higher Cryptococcus count was observed in the VPSWOV group than in the VPSV group via India ink staining tests. There was no significant difference found in CSF cell counts or protein, glucose or chloride levels between the two groups. When compared with pre-operation levels, we found that the CSF pressure in both groups and fungal count in the VPSV group were significantly decreased within 1 week after the operation. Moreover, we presented the status when patients were discharged from hospital and when they returned to hospital after 1 month. Interestingly, we see similar results at these two observation time points. There were significantly decreased ICH and fungal loads after VPS in both the VPSV and VPSWOV groups. VPS helped to reduce ICH and fungal load. However, our results suggest patients would have poor outcomes if they have a serious underlying disease.

Continuous ICH, in CM patients both with and without ventriculomegaly, can lead to irreversible neurological complications and increase morbidity and mortality. Failure to address the consequences of ICH would be significantly detrimental. If the initial treatment, even combined with appropriate antifungal therapy, still fails to control ICH, VPS could be an effective choice. Our study has shown that CSF pressure and Cryptococcus counts were significantly decreased after VPS, and headache could continue to improve.

## Conclusions

In summary, our data suggest that early placement of a VPS is helpful in decreasing uncontrollable ICH and fungal overload, regardless of the ventriculomegaly status of non-HIV CM patients. The VPS could not only relieve the symptoms of ICH, but could also improve the clinical features of CM patients. Therefore, early diagnosis and early use of VPS in CM patients, before the onset of severe neurological deficit symptoms, could be beneficial and essential. We look forward to future clinical studies to confirm this result.

### Limitations

Our study has some limitations. Due to the exclusion of other surgical methods and some missing data at follow-up, this study had a limited sample size. Further research with a multicentre study and a larger samples size will be more convincing.

## Abbreviations

5FC: Flucytosine
AIHA: Autoimmune haemolytic anaemia
AMB: Amphotericin B
CM: Cryptococcal meningitis
CSF: Cerebrospinal fluid
CT: Computed tomography
EVD: External ventricular drainage
Fluc: Fluconazole
ICH: Intracranial hypertension
ICP: Intracranial pressure
LP: Lumbar punctures
MRI: Magnetic resonance imaging
VPS: Ventriculoperitoneal shunts
VPSV: VPS with ventriculomegaly
VPSWOV: VPS without ventriculomegaly

## References

[1] In: Rapid Advice: Diagnosis, Prevention and Management of Cryptococcal Disease in HIV-Infected Adults, Adolescents and Children*.* edn. Geneva; 2011. 26110194

[2] Panackal AA, Wuest SC, Lin YC, Wu T, Zhang N, Kosa P, Komori M, Blake A, Browne SK, Rosen LB, Hagen F, Meis J, Levitz SM, Quezado M, Hammoud D, Bennett JE, Bielekova B, Williamson PR (2015). Paradoxical immune responses in non-HIV Cryptococcal meningitis. PLoS Pathog.

[3] Pyrgos V, Seitz AE, Steiner CA, Prevots DR, Williamson PR (2013). Epidemiology of cryptococcal meningitis in the US: 1997-2009. PLoS One.

[4] Ou XT, Wu JQ, Zhu LP, Guan M, Xu B, Hu XP, Wang X, Weng XH (2011). Genotypes coding for mannose-binding lectin deficiency correlated with cryptococcal meningitis in HIV-uninfected Chinese patients. J Infect Dis.

[5] Saag MS, Graybill RJ, Larsen RA, Pappas PG, Perfect JR, Powderly WG, Sobel JD, Dismukes WE (2000). Practice guidelines for the management of cryptococcal disease. Clin Infect Dis.

[6] Vidal JE, Gerhardt J, Peixoto de Miranda EJ, Dauar RF, Oliveira Filho GS, Penalva de Oliveira AC, Boulware DR (2012). Role of quantitative CSF microscopy to predict culture status and outcome in HIV-associated cryptococcal meningitis in a Brazilian cohort. Diagn Microbiol Infect Dis.

[7] Liliang PC, Liang CL, Chang WN, Chen HJ, Su TM, Lu K, Lu CH (2003). Shunt surgery for hydrocephalus complicating cryptococcal meningitis in human immunodeficiency virus-negative patients. Clin Infect Dis.

[8] de Vedia L, Arechavala A, Calderon MI, Maiolo E, Rodriguez A, Lista N, Di Virgilio E, Cisneros JC, Prieto R (2013). Relevance of intracranial hypertension control in the management of Cryptococcus neoformans meningitis related to AIDS. Infection.

[9] Cherian J, Atmar RL, Gopinath SP. Shunting in cryptococcal meningitis. Journal of neurosurgery. 2016;125:177–86. 10.3171/2015.4.JNS15255 26517766

[10] Liu L, Zhang R, Tang Y, Lu H (2014). The use of ventriculoperitoneal shunts for uncontrollable intracranial hypertension in patients with HIV-associated cryptococcal meningitis with or without hydrocephalus. Bioscience trends.

[11] Park MK, Hospenthal DR, Bennett JE (1999). Treatment of hydrocephalus secondary to cryptococcal meningitis by use of shunting. Clin Infect Dis.

[12] Corti M, Priarone M, Negroni R, Gilardi L, Castrelo J, Arechayala AI, Messina F, Franze O (2014). Ventriculoperitoneal shunts for treating increased intracranial pressure in cryptococcal meningitis with or without ventriculomegaly. Rev Soc Bras Med Trop.

[13] Liliang PC, Liang CL, Chang WN, Lu K, Lu CH (2002). Use of ventriculoperitoneal shunts to treat uncontrollable intracranial hypertension in patients who have cryptococcal meningitis without hydrocephalus. Clin Infect Dis.

[14] Bahr NC, Boulware DR (2014). Methods of rapid diagnosis for the etiology of meningitis in adults. Biomark Med.

[15] Perfect JR, Dismukes WE, Dromer F, Goldman DL, Graybill JR, Hamill RJ, Harrison TS, Larsen RA, Lortholary O, Nguyen MH, Pappas PG, Powderly WG, Singh N, Sobel JD, Sorrell TC (2010). Clinical practice guidelines for the management of cryptococcal disease: 2010 update by the infectious diseases society of america. Clin Infect Dis.

[16] Lee YC, Wang JT, Sun HY, Chen YC (2011). Comparisons of clinical features and mortality of cryptococcal meningitis between patients with and without human immunodeficiency virus infection. J Microbiol Immunol Infect.

[17] Liao CH, Chi CY, Wang YJ, Tseng SW, Chou CH, Ho CM, Lin PC, Ho MW, Wang JH (2012). Different presentations and outcomes between HIV-infected and HIV-uninfected patients with Cryptococcal meningitis. J Microbiol Immunol Infect.

[18] Bicanic T, Brouwer AE, Meintjes G, Rebe K, Limmathurotsakul D, Chierakul W, Teparrakkul P, Loyse A, White NJ, Wood R, Jaffar S, Harrison T (2009). Relationship of cerebrospinal fluid pressure, fungal burden and outcome in patients with cryptococcal meningitis undergoing serial lumbar punctures. AIDS.

[19] Husain M, Jha DK, Rastogi M (2005). Angiographic catheter: unique tool for neuroendoscopic surgery. Surg Neurol.

[20] Iwashita T, Kitazawa K, Koyama J, Nagashima H, Koyama T, Tanaka Y, Hongo K (2005). A saccular-like dissecting aneurysm of the anterior cerebral artery that developed 2 years after an ischemic event. Surg Neurol.

[21] Petrou P, Moscovici S, Leker RR, Itshayek E, Gomori JM, Cohen JE (2012). Ventriculoperitoneal shunt for intracranial hypertension in cryptococcal meningitis without hydrocephalus. J Clin Neurosci.

[22] Wang H, Ling C, Chen C, He HY, Luo L, Ning XJ (2014). Evaluation of ventriculoperitoneal shunt in the treatment of intracranial hypertension in the patients with cryptococcal meningitis: a report of 12 cases. Clin Neurol Neurosurg.

[23] Stevens DA, Denning DW, Shatsky S, Armstrong RW, Adler JD, Lewis BH (1999). Cryptococcal meningitis in the immunocompromised host: intracranial hypertension and other complications. Mycopathologia.

[24] Lee SC, Casadevall A (1996). Polysaccharide antigen in brain tissue of AIDS patients with cryptococcal meningitis. Clin Infect Dis.

